# Late Gadolinium Enhancement Cardiovascular Magnetic Resonance Assessment of Substrate for Ventricular Tachycardia With Hemodynamic Compromise

**DOI:** 10.3389/fcvm.2021.744779

**Published:** 2021-10-26

**Authors:** John Whitaker, Radhouene Neji, Steven Kim, Adam Connolly, Thierry Aubriot, Justo Juliá Calvo, Rashed Karim, Caroline H. Roney, Brendan Murfin, Carla Richardson, Stephen Morgan, Tevfik F. Ismail, James Harrison, Judith de Vos, Maurice C. G. Aalders, Steven E. Williams, Rahul Mukherjee, Louisa O'Neill, Henry Chubb, Cory Tschabrunn, Elad Anter, Luigi Camporota, Steven Niederer, Sébastien Roujol, Martin J. Bishop, Matthew Wright, John Silberbauer, Reza Razavi, Mark O'Neill

**Affiliations:** ^1^School of Biomedical Engineering and Imaging Sciences, King's College, London, United Kingdom; ^2^Siemens Healthcare, Frimley, United Kingdom; ^3^Abbott Medical, St Paul, MN, United States; ^4^Brighton and Sussex University Hospitals NHS Trust, Brighton, United Kingdom; ^5^Guy's and St Thomas' NHS Foundation Trust, London, United Kingdom; ^6^Department of Biomedical Engineering and Physics, Amsterdam UMC, University of Amsterdam, Amsterdam, Netherlands; ^7^Centre for Cardiovascular Science, University of Edinburgh, Edinburgh, United Kingdom; ^8^Division of Cardiovascular Medicine, University of Pennsylvania, Philadelphia, PA, United States; ^9^Cleveland Clinic, Cleveland, OH, United States

**Keywords:** ventricular tachycardia, late gadolinium enhancement, cardiovascular magnetic resonance, mechanical circulatory support, venous-arterial extra corporeal membrane oxygenation (VA-ECMO)

## Abstract

**Background:** The majority of data regarding tissue substrate for post myocardial infarction (MI) VT has been collected during hemodynamically tolerated VT, which may be distinct from the substrate responsible for VT with hemodynamic compromise (VT-HC). This study aimed to characterize tissue at diastolic locations of VT-HC in a porcine model.

**Methods:** Late Gadolinium Enhancement (LGE) cardiovascular magnetic resonance (CMR) imaging was performed in eight pigs with healed antero-septal infarcts. Seven pigs underwent electrophysiology study with venous arterial-extra corporeal membrane oxygenation (VA-ECMO) support. Tissue thickness, scar and heterogeneous tissue (HT) transmurality were calculated at the location of the diastolic electrograms of mapped VT-HC.

**Results:** Diastolic locations had median scar transmurality of 33.1% and a median HT transmurality 7.6%. Diastolic activation was found within areas of non-transmural scar in 80.1% of cases. Tissue activated during the diastolic component of VT circuits was thinner than healthy tissue (median thickness: 5.5 mm vs. 8.2 mm healthy tissue, *p* < 0.0001) and closer to HT (median distance diastolic tissue: 2.8 mm vs. 11.4 mm healthy tissue, *p* < 0.0001). Non-scarred regions with diastolic activation were closer to steep gradients in thickness than non-scarred locations with normal EGMs (diastolic locations distance = 1.19 mm vs. 9.67 mm for non-diastolic locations, *p* < 0.0001). Sites activated late in diastole were closest to steep gradients in tissue thickness.

**Conclusions:** Non-transmural scar, mildly decreased tissue thickness, and steep gradients in tissue thickness represent the structural characteristics of the diastolic component of reentrant circuits in VT-HC in this porcine model and could form the basis for imaging criteria to define ablation targets in future trials.

## Introduction

Viable myocytes within and on the border of dense scar display abnormal electrophysiological characteristics that promote reentrant arrhythmias (1–3). In most clinical VT ablation procedures, electrophysiological criteria alone are normally used to identify arrhythmogenic tissue (4–6). Even with a combination of ablation of clinical VT identified through activation and entrainment mapping and pace-mapping as well as substrate-based ablation (7), medium term outcomes remain modest with up to 50% of patients experiencing a recurrence of VT within a year of ablation (8).

When imaged *ex-vivo* at the near-cellular level, late-gadolinium enhanced (LGE) cardiovascular magnetic resonance (CMR) accurately identifies peri-infarct heterogeneous tissue (HT) as regions of intermediate signal intensity (ISI) (9). This tissue represents the critical substrate responsible for post-MI reentrant VT (10). These observations have been extended to *in-vivo* imaging where it has been reported that regions of ISI may also display abnormal electrophysiological properties (11, 12) described as the peri-infarct borderzone (BZ). This suggests that *in-vivo* LGE-CMR may offer a low-risk option for generating a 3D assessment of substrate that may have utility in guiding substrate-based VT ablation.

Pre-procedural cross-sectional imaging has been proposed as a non-invasive approach that may facilitate more comprehensive arrhythmogenic substrate identification and ablation (13). Effective substrate identification, either using electrophysiological or structural criteria, may be of particular importance in the context of the growing population of patients presenting with VT with hemodynamic compromise (VT-HC) (14, 15), in which only a substrate-guided approach is usually possible (6).

We hypothesized that under idealized experimental conditions the quality of LGE-CMR could be optimized in order provide accurate anatomic information about the tissue substrate for VT-HC. We aimed to characterize the tissue substrate responsible for VT-HC using high-resolution *in-vivo* 3D LGE-CMR and acquiring high-density activation mapping under hemodynamic support to identify the location of diastolic electrograms during post-MI VT-HC in a chronic porcine infarct model.

## Materials and Methods

Animal studies complied with French law and were performed at the Institut de Chirurgie Guidée par l'image (IHU), Strasbourg, France. The experimental protocol was approved by the local and national institutional animal care and ethics committee. Eight domestic pigs underwent a 180 minute balloon occlusion of the mid left anterior descending (LAD) artery to create experimental ischemia-reperfusion myocardial infarction (MI) as previously described (16). Seven weeks following MI each pig underwent late-gadolinium enhanced (LGE) cardiovascular magnetic resonance (CMR) imaging. One week later each pig underwent electrophysiology study with prophylactic hemodynamic support during which VT was induced and assessed using high-density activation mapping.

## Cardiovascular Magnetic Resonance Imaging Data Acquisition

All imaging was performed on 1.5T scanner (MAGNETOM Aera, Siemens Healthineers, Erlangen, Germany) with an 18-channel body matrix coil and a 32-channel spine coil.

### Clinical Standard 2D Late Gadolinium Enhanced Cardiovascular Magnetic Resonance Imaging

Ten min after 0.1 mmol/kg gadobutrol (Gadovist, Bayer Plc, Reading, UK) bolus, 2D LGE-CMR imaging was acquired. Inversion-time (TI) scout was acquired in a single mid ventricular short axis slice [free breathing, balanced steady state free precession (bSSFP); linear k-space reordering; echo time/repetition time/flip angle (TE/TR/α): 1.11 ms/2.6 ms/30°; slice thickness: 8 mm; in plane resolution: 1.8 x 1.8 mm^2^; field of view (FOV): 340 x 276 mm^2^]. Clinical standard 2D LGE-CMR imaging was acquired using an inversion recovery (IR) sequence with phase-sensitive IR (PSIR) reconstruction (bSSFP; linear k-space reordering; TE/TR/α: 1.21 ms/3 ms/45°; slice thickness: 6 mm; in plane resolution: 1.4 x 1.4 mm^2^; FOV: 360 x 281 mm^2^). Fifteen min after Gd bolus, 0.0011 mmol/kg/min Gd infusion was commenced. TI-scout was repeated at regular intervals following Gd bolus and during infusion.

### 3D Late Gadolinium Enhanced Cardiovascular Magnetic Resonance Imaging

Isotropic navigator-gated ECG-triggered 3D IR sequence was acquired in the mid-diastolic phase as identified from cine imaging (fat saturation prepared; bSSFP; coronal orientation; linear k-space reordering; TE/TR/α: 1.58ms/3.6ms/90°; gating window = 7 mm; parallel imaging using GRAPPA with acceleration factor 2; resolution 1.2 x 1.2 x 1.2 mm^3^; FOV: 400 x 257 x 96 mm^3^; 2 R-R interval ECG triggering) with full ventricular coverage. Subjective and objective parameters of image quality were compared between 2D and 3D LGE CMR imaging (further details in Supplementary Figure 1).

## Cardiovascular Magnetic Resonance Imaging Data Analysis

### Image Segmentation

Each 3D LGE-CMR scan was manually segmented by a single observer within Seg3D2 [University of Utah, Utah, USA, https://www.sci.utah.edu/cibc-software/seg3d.html]. Based on previously reported analysis of LGE-CMR imaging from patients undergoing VT ablation (17), the segmented myocardium was thresholded for scar signal intensity (SI_scar_) at 60% and intermediate SI (SI_intermediate_) at 40% of the highest SI within the segmentation.

### Tissue Thickness and Scar Transmurality Assessment

Field lines between the endocardial and epicardial surfaces were derived by solving the Laplace equation as previously described (18). Briefly, the endocardial and epicardial surfaces were tagged. To calculate the distance from endocardial to epicardial surface, the Laplace equation (∇^2^u = 0) was solved with Dirichlet boundary conditions assigned at the endocardial (u = 0) and epicardial (u = 1) surfaces. Wall thickness was evaluated as the total length of the continuous path from the endocardium to the epicardium when moving orthogonally between adjacent iso-potential surfaces. Tissue thickness gradients were assessed using a radial basis function to identify the gradient in tissue thickness (further details in Supplementary Materials). Transmurality of scar and HT was defined as the proportion of nodes along the path between endocardium and epicardium corresponding to each tissue type. The distance of each endocardial node to the nearest neighboring node assigned as heterogeneous tissue (HT) was automatically calculated.

Segmentations were processed to generate a surface mesh containing endocardial and epicardial surfaces, scar and aorta in the Digital Image Fusion (DIF) format for import into the Precision Electroanatomic Mapping System (EAMS, Abbott, St Paul, MN, USA) within which they were registered with the EAMS geometry, as described below.

### Electrophysiology Study and Hemodynamic Support

Electrophysiology procedures were conducted 8 weeks after MI under general anesthetic (further details in Supplementary Materials) using the Precision electro-anatomic mapping system (EAMS, Abbott, Chicago, IL).

Prior to the EP study pigs were established on venous-arterial extra-corporeal membrane oxygenation (VA-ECMO) via the left femoral artery and vein and hemodynamic support was prophylactically instituted using a Maquet Cardiohelp machine (Maquet Getinge group, Rastatt, Germany, see Supplementary Figure 1). Venous and arterial access was established for the EP study and hemodynamic monitoring/drug administration. Hemodynamic compromise (HC) was defined as a rhythm associated with a mean arterial pressure (MAP) below 50 mmHg or a pulse pressure lower than 20 mmHg. During rhythms not associated with HC, VA-ECMO circuit flow was reduced to 0.5 L/min. During rhythms associated with HC, VA-ECMO flow ranged between 0.5 and 1 L to 4 L depending on hemodynamic status to maintain a MAP of 65–70 mmHg. Further details are provided in the Supplementary Materials.

A sensor-enabled Abbott FlexAbility™ ablation catheter was advanced to the aorta via the right femoral artery and used to acquire aortic root and coronary ostia geometry prior to gaining retrograde left ventricular (LV) access across the aortic valve. A multipolar mapping catheter [HD Grid™ or LiveWire™ duo-deca (Abbott, Chicago, IL)] was advanced through an Agilis sheath via the aorta to acquire LV endocardial geometry.

LV endocardial activation maps were acquired while pacing from the RV apex at 500 ms and then 300 ms.

An attempt was made to induce ventricular tachycardia (VT) using an adapted Wellen's VT stimulation protocol (19) with up to four extra-stimuli from right and then left ventricular sites. In the event that no VT was induced, the non-selective beta agonist isoproterenol was commenced as an infusion at an initial rate of 2 μg/min and up titrated to a maximum rate of infusion of 20 μg/min during which programmed electrical stimulation was repeated.

Activation maps were acquired using the Automap function within Precision™ using the initial deflection seen on any lead of the surface ECG leads as the timing reference with strict settings applied (EAMS, Abbott, St Paul, MN, USA) (Score = 85; speed limit = 10 mm/s; distance 1 mm; enhanced noise rejection: off). Each individual bipolar and unipolar EGM from the mapped VTs and maps during pacing were subsequently reviewed offline and the activation time reassigned when necessary. Following spontaneous or pace-termination, VT induction and mapping was subsequently repeated. Final activation maps were reviewed by at least two experienced electrophysiologists.

The extent of conduction block (identified as regions with tightly spaced isochrones in which > 15 ms separated adjacent activation points; in which activation on either side of tightly spaced isochrones from wave fronts moving in different directions, and in which double potentials identified in proximity to the boundary between adjacent waves of conduction) during RV pacing and during each mapped VT was estimated using the surface distance function within the EAMS.

### Co-registration of Imaging and Electrophysiological Data

After the EP study, using the re-map function within the EAMS, a surface mesh generated from the LGE-CMR scan was imported into the EAMS in the digital image fusion (DIF) format. The EAMS LV geometry was registered to the DIF model with an initial landmark-based registration with the aorta, coronary ostia and LV apex as initial fiducial landmarks, followed by surface registration using the proprietary surface registration function within the EAMS. EAMS points manually identified as having healthy EGMs during RVP_500_ and those demonstrating diastolic activation during VT were exported from the EAMS. The location of these points from the EAMS mesh were automatically mapped to nodes on the CMR derived mesh within a 1 mm radius, to allow a node-wise assessment of the structural characteristics of tissue demonstrating diastolic activation during VT and healthy tissue could be made.

### Episcopic Auto-Fluorescence Cryomicrotome Imaging

Following euthanasia, each heart was processed and then imaged using episcopic auto-fluorescence cryomicrotome imaging as previously described (20) (see Supplementary Figure 2).

### Statistical Analysis

Normality of distribution of variables was assessed using the Shapiro-Wilks test. Normally distributed continuous variables are expressed as mean ± SD, otherwise variables are reported as medians with interquartile ranges. Normally distributed data were compared using a two tailed, paired sample *t-*test. Ordinal and non-parametric continuous data were compared using a Mann-Whitney U test or Kruskal-Wallis H-test as appropriate, with pairwise comparisons performed using Dunn's (1964) procedure with a Bonferroni correction for multiple comparisons where appropriate in which case adjusted *p*-values are presented. Two-tailed values of *p* < 0.05 were considered significant. Statistical analysis was carried out in SPSS (v24, IBM Corporation, New York).

## Results

One post-MI pig died after CMR imaging and prior to EP study. The study protocol was completed in the remaining seven post-MI pigs and two control pigs who did not undergo CMR imaging. During this study all the VT that was induced was associated with hemodynamic compromise as defined above.

### Late Gadolinium Enhanced Imaging Under Contrast Steady State

**Figure 1:**
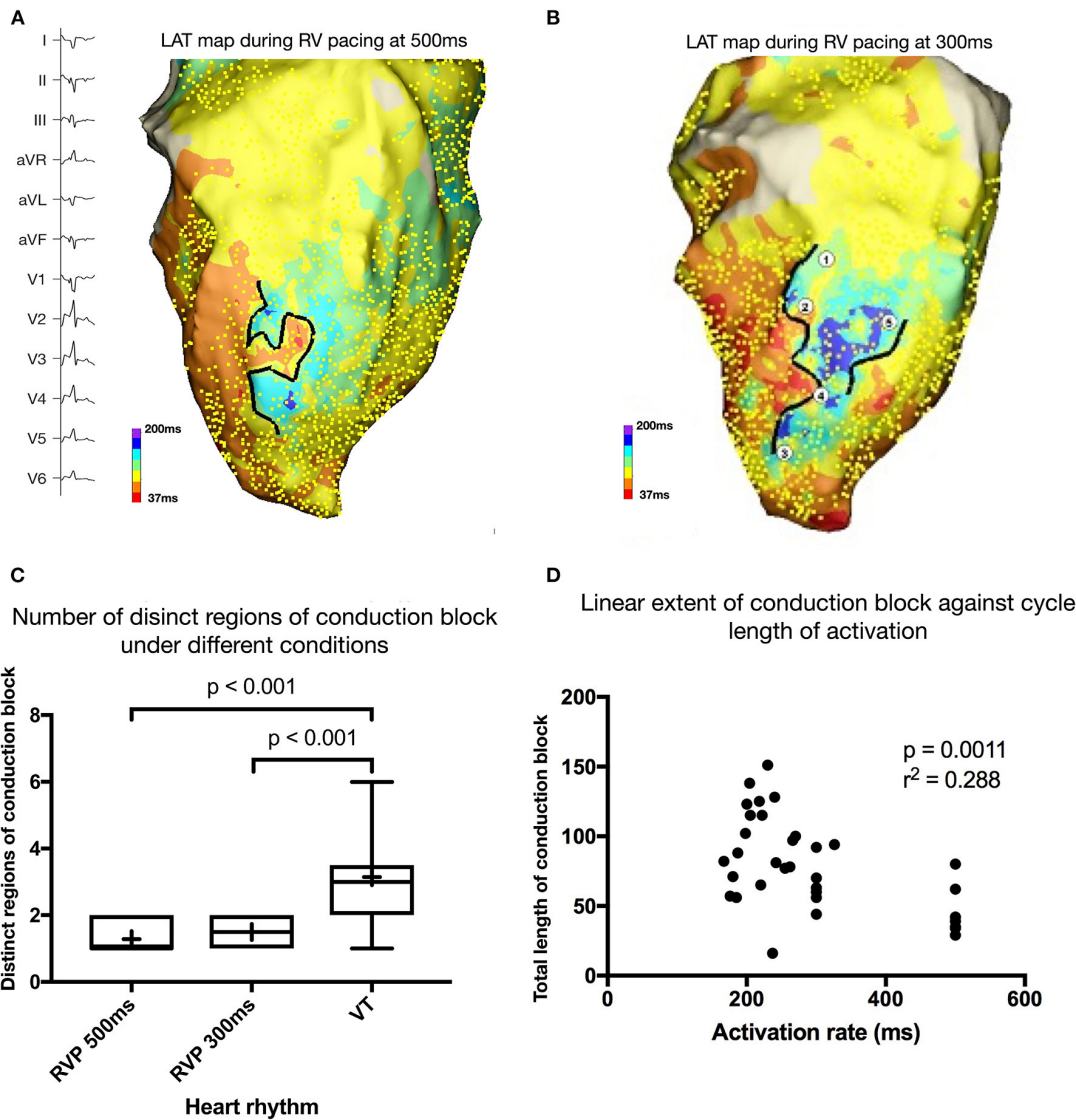
**(A)** Activation map during right ventricular (RV) pacing at 500 ms. Yellow dots mark the point at which activation time was recorded. Black line represents linear conduction block during pacing at this cycle length. **(B)** Activation map during right ventricular (RV) pacing at 300 ms. **(C)** Number of distinct regions of conduction block during different heart rhythms (RV pacing at 500 ms and 300 ms and during VT). **(D)** Scatter plot of extent of linear endocardial conduction block and activation rate during pacing or VT.

3D LGE-CMR imaging was acquired in eight animals (weight 62.6+/−3.7 kg) in the supine position under general anesthesia and CSS achieved in seven animals. In a single animal failure of the peripheral cannula interrupted the continuous Gd infusion, which prevented CSS during imaging. In the remaining seven animals 3D LGE-CMR imaging was acquired using a total Gd dose of <0.2 mmol/kg. 3D LGE-CMR was acquired in mid-diastole with a data acquisition duration of 83–104 ms, 23–42 segments, and a total imaging time of 50+/−12 min. Under conditions of CSS mean variation in TI_myocardium_ was 5.2% (±3.1%, range 2.6–9.3%) and mean variation in TI_blood_ was 9.8% (±2.1%, range 6.7–13.5%). Representative imaging examples and demonstration of CSS are shown in Figure 1. All 3D LGE-CMR imaging is available for review online (Supplementary Material).

A comparison of the image quality with clinical standard 2D LGE-CMR imaging was undertaken and is included in the Supplementary Materials.

### Induced Ventricular Tachycardia

Twenty episodes of VT-HC demonstrating a macro reentrant pattern of activation were mapped (18 complete maps, 2 incomplete) and the locations of diastolic electrograms (EGMs) assessed to be part of the reentrant circuit were identified and Labeled. A summary of the characteristics of the induced VTs is shown in Table 1. All mapped VTs were dependent on at least one region of conduction block that was not evident during RVP_500_. All VTs with a complete activation map demonstrated at least two distinct regions of conduction block.

**Table 1:** Number of tachyarrhythmias observed and mapped per animal.

**Pig**	**Number of episodes of non-sustained VT**	**Number of sustained, unmapped VTs**	**Number of mapped VTs**	**Number of episodes VF**
1	67	3	0	7
2	-	-	-	-
3	27	2	4	1
4	100	3	9	4
5	22	2	2	6
6	16	3	2	10
7	88	2	2	1
8	47	3	2	8

There was an increase in the extent of conduction block between RVP_500_ and VT (mean increase = 45 mm, 95% CI 27–63 mm, *p* < 0.001) and between RVP_300_ and VT (mean increase 23 mm, 95% CI 5–42 mm, *p* = 0.016), and a trend toward, an increase in conduction block between RVP_500_ and RVP_300_ (mean increase = 22 mm, 95% CI−1–45 mm, *p* = 0.059). Pearson correlation coefficient demonstrated a negative correlation between activation rate and total extent of conduction block (r^2^ = 0.288, *p* = 0.001). These data are illustrated in Figure 1. Entrainment mapping was attempted in a subset of the induced VT-HC but was not successful due to failure to entrain or degeneration of the VT to ventricular fibrillation (VF).

### Tissue Structure Assessment With *in-vivo* CMR at Locations With Diastolic Activation

**Figure 2:**
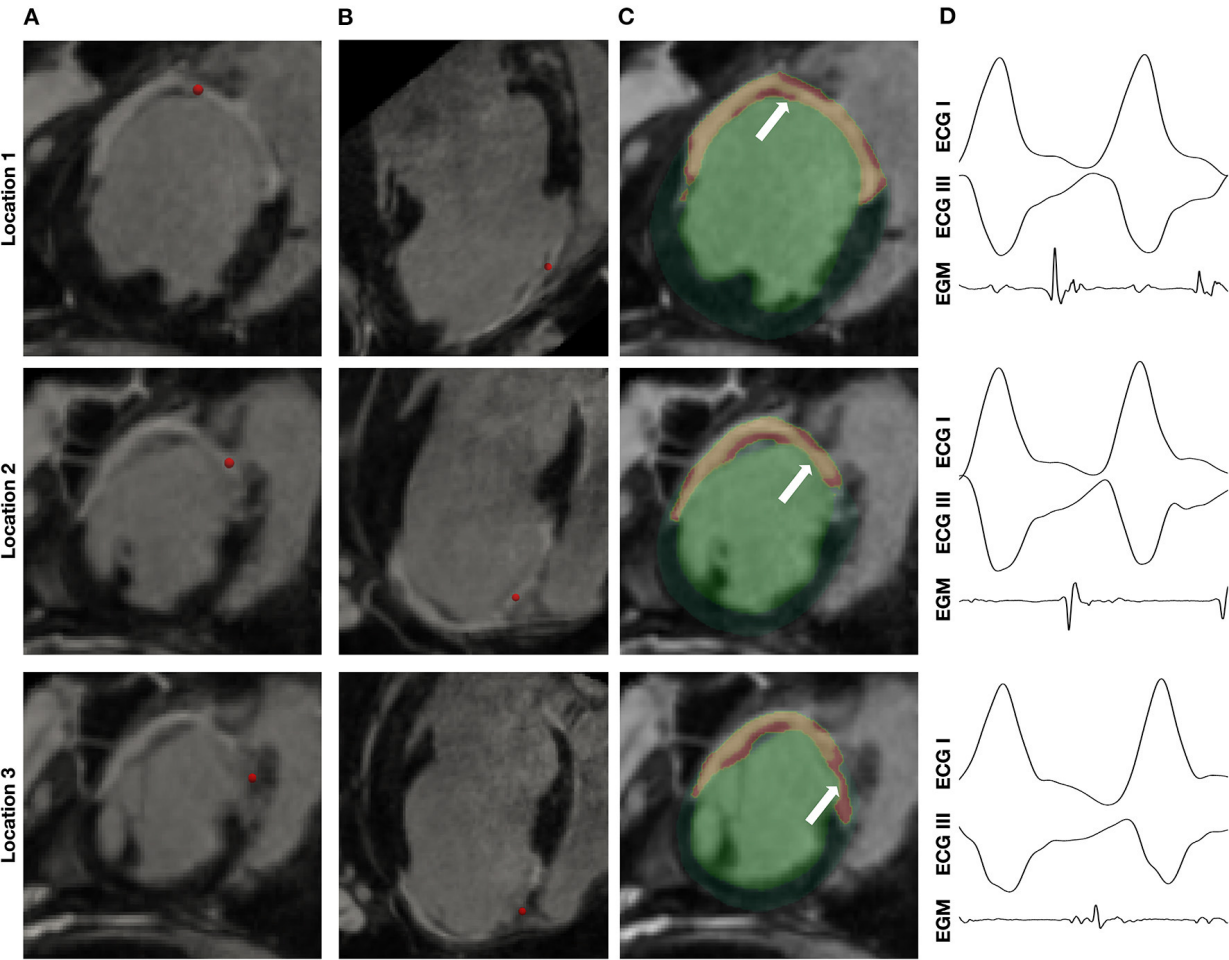
Visualization of location of diastolic EGMs on *in-vivo* CMR. Column **(A)** and column **(B)** show short axis (SAX) and long axis (LAX) multiplanar reconstruction (MPR) of *in-vivo* CMR with the location of the corresponding diastolic EGM [column **(D)**] indicated by a red sphere. Column **(C)** shows SAX MPR of *in-vivo* CMR with segmentation of scar (yellow) and heterogeneous tissue (HT) (red) superimposed.

Representative examples of diastolic EGMs recorded during VT are shown with their corresponding locations on *in-vivo* CMR in Figure 2. As shown in these examples, diastolic EGMs were visualized in regions with subendocardial HT (location 1), near-transmural scar (location 2) and pure HT (location 3). Tissue thinning is also seen in all locations.

**Figure 3:**
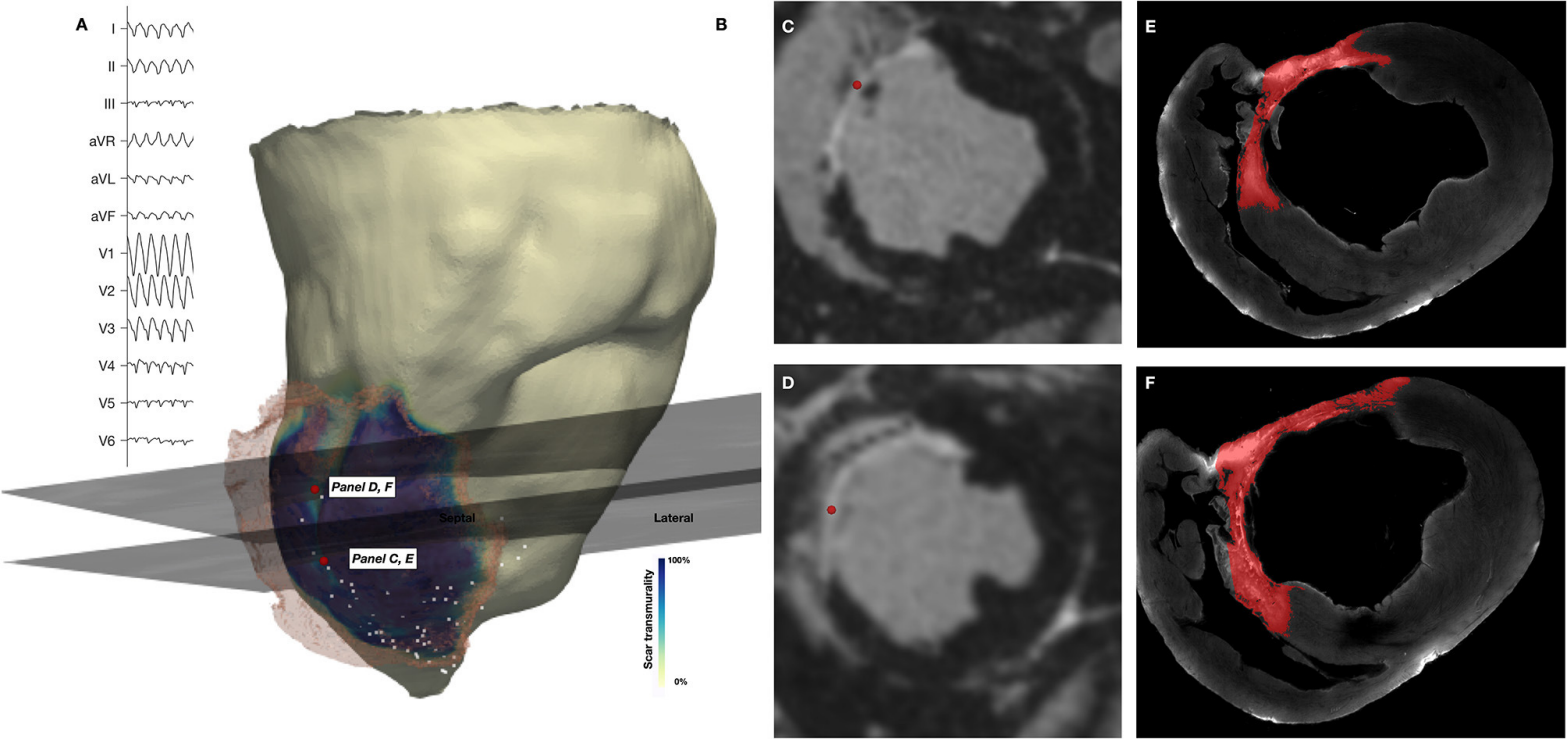
Co-localization of diastolic locations from Electroanatomic Mapping System and *in-vivo* imaging and episcopic auto-fluorescence cryomicrotome imaging. **(A)** Surface 12-lead ECG of induced VT. **(B)** *In-vivo* CMR derived shell color coded according to scar transmurality, with translucent mesh derived from scar also shown. Location of 2 diastolic EGMs is shown (red spheres) and corresponding short axis (SAX) *in-vivo* LGE CMR slice. **(C,D)** SAX slices shown in **(B)**, with location of recorded EGM (red sphere). **(E,F)** Corresponding EACI data with segmented scar (red) superimposed.

A representative example of the position of a diastolic EGM, corresponding *in-vivo* imaging and episcopic auto-fluorescence cryomicrotome imaging (EACI) data is shown in Figure 3.

**Figure 4:**
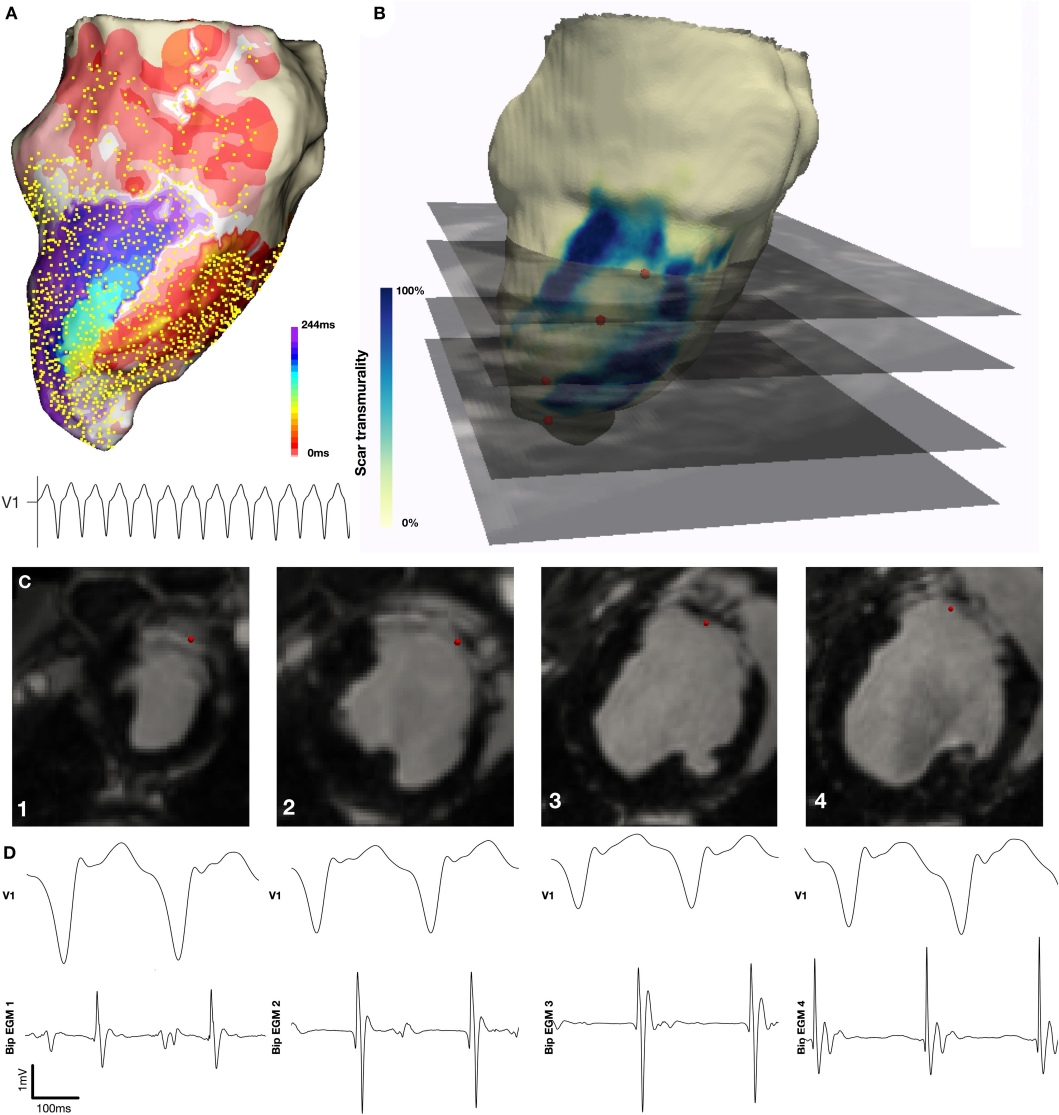
Localization of diastolic locations on *in-vivo* CMR imaging. **(A)** Activation map demonstrating re-entrant VT with a figure 8 pattern of re-entry. **(B)** CMR derived mesh color coded according to scar transmurality with location of selected diastolic EGMs indicated by red sphere and corresponding *in-vivo* LGE CMR imaging indicated. **(C)** Diastolic EGM and corresponding location on *in-vivo* CMR at four sequential points along the diastolic isthmus of this VT.

Non-enhanced tissue adjacent to regions of enhancement on *in-vivo* CMR were frequently observed in the septal area and corresponded to regions where muscle fibers traversing the RV cavity, including the moderator band, inserted into the septum. An example of this is shown in Figure 4, in which a corridor of preserved myocardium surrounded by dense scar forms the principal path of the diastolic isthmus of one VT, which in this case is approximately aligned with the direction of the long axis of the left ventricle.

**Figure 5:**
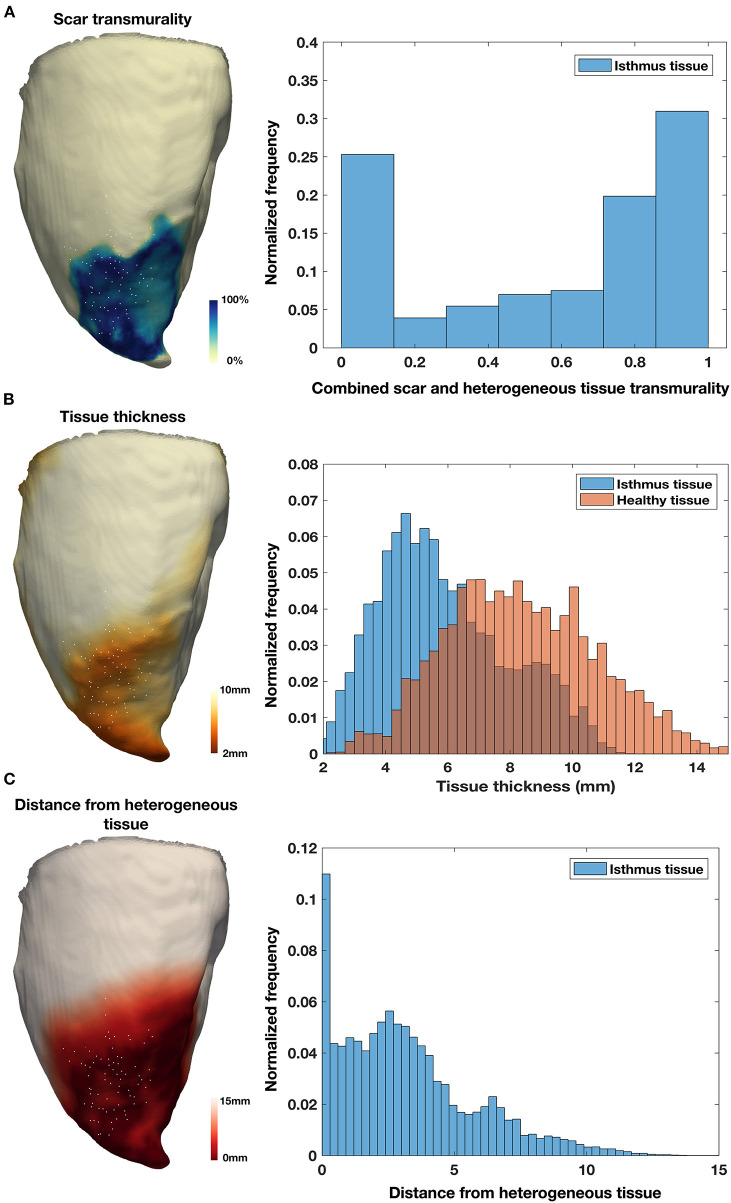
Tissue characteristics of diastolic locations. **(A)** Example of LGE CMR derived endocardial shell color coded according to scar transmurality and demonstrating antero-septal infarction. Histogram shows combined scar/heterogeneous tissue (HT) transmurality pooled from all locations activated during diastole in all pigs. **(B)** Example of LGE CMR derived endocardial shell color coded according to tissue thickness. Histogram shows tissue thickness pooled from all diastolic locations (blue) in all pigs compared with tissue thickness at the location of normal EGMs pooled from all pigs (red). **(C)** Example of LGE CMR derived endocardial shell color coded according to distance from HT. Histogram shows distance from HT pooled from all diastolic locations in six pigs.

The structural characteristics of locations at which diastolic EGMs occurred during the observed VTs were systematically examined and compared with locations at which normal EGMs were recorded. The normal EGMs were all recorded at positions with 0% scar/HT transmurality. Diastolic locations had a median scar transmurality of 33.1%, [IQR 0–77.3%, mean 37.4(±36.3) %, *p* < 0.001] and a median HT transmurality 7.6% [IQR 0–30.3%, mean 18.3(±22.9)% *p* < 0.001]. The majority (80.1%) of diastolic locations were found within areas with non-transmural scar or HT. Tissue activated in the diastolic component of VT circuits was thinner than healthy tissue (median thickness tissue with diastolic activation = 5.5 mm vs. 8.2 mm healthy tissue, *p* < 0.001) and was closer to HT (median distance from HT diastolically activated tissue 2.8 mm vs. 11.4 mm healthy tissue, *p* < 0.001). Of those diastolic locations that were in regions without scar or HT, all were within 15 mm of HT and 89% lay within 10 mm of HT. These results are illustrated in Figure 5.

### Non-scarred Regions With Diastolic Activation During Ventricular Tachycardia

**Figure 6:**
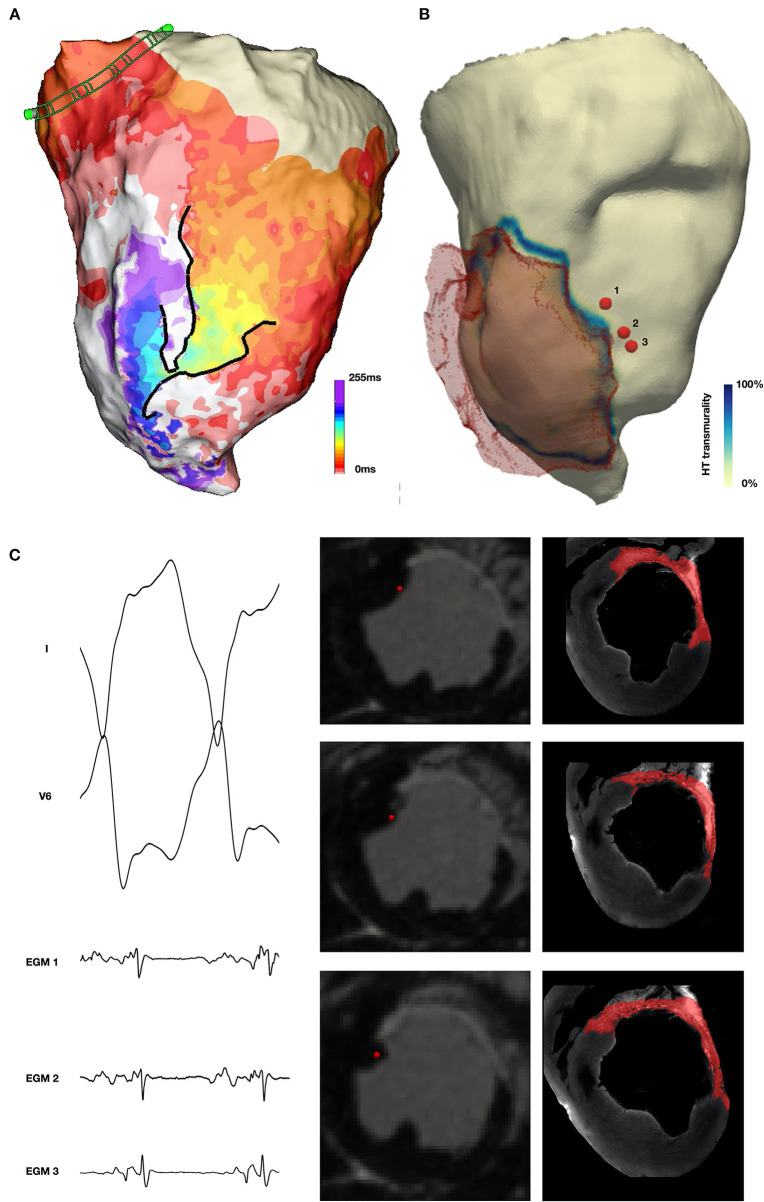
Non-scarred tissue demonstrating diastolic activation during VT. **(A)** Activation map during VT demonstrating diastolic activation during VT (yellow to purple) with a converging pattern of activation toward a narrow channel (green) which subsequently widens prior to multiple systolic breakouts (red). **(B)** *In-vivo* CMR derived shell with translucent mesh of scar overlaid and color coded according to HT transmurality. The location of 3 early diastolic EGMs is indicated by red spheres. **(C)** Early diastolic EGM, short axis (SAX) *in-vivo* CMR and corresponding EACI imaging at locations 1, 2 and 3 demonstrating location of EGMs in tissue proximal to scar, but without enhancement in the wall at this location.

Approximately 20% of diastolic points had 0% scar/HT on *in-vivo* CMR. As noted, all regions activated during diastole during VT-HC were within 15 mm of HT. An example of EGMs recorded from a reentrant VT-HC is shown in Figure 6. In this example, early diastolic activation is identified within non-scarred tissue, before the wavefront continues into a region demonstrating non-transmural scar in which the remainder of the isthmus is located. On the corresponding *in-vivo* CMR and EACI data the EGMs are located adjacent to a sharp gradient in tissue thickness just outside the thinned scar region. When all locations without scar were considered, locations with diastolic activation were closer to steep gradients in tissue thickness than non-diastolic locations (diastolic locations distance = 1.2 mm (IQR 0–3.6 mm) vs. 9.7 mm (IQR 2.9–18.6 mm) for non-diastolic locations, *p* < 0.001). When diastolic locations were classified according to whether they demonstrated early, mid or late activation within the diastolic window, defined as being from the end of the QRS complex on the surface ECG, the median distance to regions of steep gradient in tissue thickness was lowest at late-diastolic sites (distance = 1.5 mm), followed by early-diastolic sites (distance = 2.8 mm) and greatest at mid-diastolic sites (distance = 3.9 mm, *p* < 0.001 for group differences and *p* < 0.001 for all pairwise comparisons).

## Discussion

In a chronic porcine infarct model high resolution 3D LGE-CMR acquired during contrast steady state can be used to define the structural characteristics of components of post-infarct reentrant VT circuits. Our data demonstrate that the majority of diastolic locations in this model of VT-HC are located in regions with non-transmural (<95%) scar/HT with lower tissue thickness than healthy tissue. In addition, ~20% of diastolic points during VT-HC are in non-scarred tissue that is adjacent to steep gradients in tissue thickness.

### Late-Gadolinium Enhanced Imaging Under Conditions of Contrast Steady State

Establishing CSS was technically feasible and resulted in stable TI_myocardium_ for prolonged periods during image acquisition. For high-resolution 3D imaging sequences used in previous imaging-supported VT ablation studies, TI_myocardium_ drift during acquisition routinely necessitated 30–80 ms being added to the TI_myocardium_ prior to the start of a 3D acquisition, which may be of up to 29 min in duration (21–23). In the present data, drift in TI_myocardium_ across the course of an extended acquisition duration was always <10% and the mean was 5.2%, corresponding to an average change in TI_myocardium_ of approximately 12 ms. In clinical practice, establishing CSS for 3D image acquisition even during scans of routine duration would minimize the drift in TI_myocardium_ and may be helpful for optimizing image quality.

The non-standard approach to contrast delivery has not been robustly validated in this study. However, it is noted that the strategy of continuous contrast infusion, on which the protocol for the current study was based, has been validated against histological samples during extra-cellular volume mapping for the assessment of fibrosis (24). There is no consensus regarding the optimal method for thresholding LGE-CMR for scar (25), and the optimal strategy may depend on the contrast administration protocol used during imaging. The Full Width-Half Maximum technique represents a strategy with robust histological validation (26), however, clinical studies have suggested that thresholding for dense scar at 60% of the maximum SI best identifies electrophysiologically relevant left ventricular substrate. This consideration led to the current strategy being chosen for this study (17). The current study does address the unresolved issue of the optimal thresholding strategy for LGE-CMR.

The 3D LGE-CMR imaging in this study was acquired with a FA of 90° which likely resulted in reduced blood-scar contrast due to the higher T2 weighting that resulted (see Supplementary Figure 3). While in this study we did not feel low contrast between blood pool and scar prohibited confident identification of the blood-myocardial interface, the differentiation of endocardial scar from the blood pool could be improved in subsequent studies through using a lower flip angle. We note that this issue is encountered to some degree in all bright-blood LGE sequences and represents a motivation to the development of dark blood sequences to overcome this effect (27).

### Transmurality of Scar

The calculated transmurality of scar in locations with diastolic activation reported here is lower than has been reported previously. In previous reports, mean scar transmurality at VT isthmus sites assessed using 2D 1.4 x 1.4 x 8 mm^3^ LGE-CMR with scar segmented according to a Full-width at half-maximum (FWHM) threshold was 60 ± 38% (28) or using a similar 2D LGE-CMR imaging 66 ± 22%, which rose to 76 ± 16% at sites of concealed entrainment and to 70 ± 21% at termination sites (29). The use of 2D imaging, bolus contrast administration and different image analysis protocols used in previous experiments is likely to contribute to observed differences in scar transmurality. In addition, there may be mechanistic differences between the VT-HC described here and VTs studied previously, both of which included at least some hemodynamically tolerated VTs. Early reports of hemodynamically tolerated scar-mediated VT often localized the isthmus to within a thin walled LV aneurysm, which would likely be identified on CMR imaging as transmural scar (30, 31). In contrast, the re-entrant VT-HC observed in this study displayed a greater dependence on functional rather than fixed conduction block, suggesting that the structural substrate of VT-HC may be distinct from slower and hemodynamically tolerated VTs. In addition, the use of high-resolution imaging and the measures taken to minimize artifact in this report may have contributed to an increased sensitivity for the identification of tissue with preserved viability and reduced the calculated scar transmurality at sites with diastolic activation.

### Tissue Thickness

Tissue thickness has been demonstrated to be a sensitive structural marker for arrhythmogenic tissue in the post-MI LV when assessed using coronary computed tomographic angiography (CCTA) (32). Among patients undergoing catheter ablation of drug refractory post-MI scar-related VT, 98% have been reported to demonstrate wall thinning on CCTA (32). As well as demonstrating a strong relationship between low voltage regions and local abnormal ventricular activations (LAVA), 89% of RF termination sites were located within the imaging substrate [defined as regions of wall thinning (tissue thickness <5 mm) or severe wall thinning (tissue thickness <2 mm)], with the majority of these located within 10 mm of the margin. The data presented here also indicate that regions of diastolic activation during VT-HC tend to be thinner than healthy tissue. However, examination of the histograms shown in Figure demonstrates that there is significant overlap between the tissue thickness assessed with diastolic activation and healthy tissue, and therefore tissue thickness alone would not adequately differentiate regions of diastolic activation during VT from healthy tissue. In the present study, the shorter VT cycle length and dependence of the VTs on functional rather than fixed anatomical conduction block, as might be expected in an aneurysm demonstrating severe wall thinning, suggests a possible explanation for why diastolic activation during VT was observed in tissue with a wider range of thicknesses than in previous reports which have used CT imaging.

### Non-enhancing Tissue Activated During Diastole in VT

Approximately 20% of diastolic locations present were found in locations with no scar/HT identified on *in-vivo* imaging. This tissue was located in close proximity to HT and adjacent to steep gradients in tissue thickness. The diastolic activation of such tissue has not been previously demonstrated in the reentrant paths of hemodynamically tolerated VT. This tissue may include regions of tissue with microscopic fibrosis that occurs adjacent to scar and that is not identified by *in-vivo* LGE-CMR imaging but is likely to demonstrate distinct electrophysiological properties that promote its participation in VT-HC. Intrinsic tissue anisotropy at the scar interface, which may be enhanced in diseased ventricular tissue (33), is also likely to affect conduction behavior in this region. In canine models of scar related re-entry, it is established that the arc of functional conduction block responsible for the initiation of reentry localizes to regions of sharp gradients in tissue thickness and previous experiments have demonstrated that conduction velocity during pacing in this model is slower in regions of steep gradients in tissue thickness (34). In addition, the lowest conduction velocity (CV) in the re-entrant circuits of VTs in the same model may be found at exit sites (35). A wave of depolarization slows when transitioning from a small to a larger body of tissue due to the dispersion of the small source transmembrane current to a large number of downstream cells (source-sink mismatch) (36) and due to wavefront curvature that is a consequence of the geometric expansion of tissue encountered by a propagating wavefront. These observations suggest a mechanistic explanation for the diastolic activation of non-scarred tissue adjacent to steep gradients in tissue thickness in the reentrant path of VT-HC defined by functional conduction block, and the greatest proximity of the late diastolic component to these areas.

### Unique Anatomical Characteristics of the Isthmus

In this study, tissue with scar/HT transmurality and tissue thickness across a wide range has been identified as activated during the diastolic component of the reentrant VT-HC studied. In a previous study of porcine post-MI VT-HC (10), despite high-resolution *ex-vivo* CMR (0.4 x 0.4 x 0.4 mm^3^), anatomic features distinguishing HT harboring diastolic activation during VT from non-participating HT were not identified. At a histological level, structural differences exist between regions harboring a critical diastolic isthmus that distinguish this tissue from other HT (37). However, since it was impossible to identify these differences using high resolution *ex-vivo* CMR, it is extremely unlikely that such arrhythmogenic HT would be distinguishable from non-arrhythmogenic HT with CMR at current *in-vivo* resolution. The tissue in which the diastolic isthmus of VT-HC is expected to be located has been characterized in this model, however the current data does not indicate that within regions of non-transmural scar/HT, arrhythmogenic and non-arrhythmogenic substrate may be differentiated.

### Limitations

Activation mapping alone was used to characterize the VT-HC circuits in this study. Entrainment mapping to confirm participation of the regions with diastolic activation was attempted but not routinely achieved and this represents a limitation of the presented data. The registration of electrophysiology and imaging data represents a major challenge when attempting to establish the structural basis for observed electrophysiological phenomena. Despite meticulous care in the registration between LGE-CMR and EAMS data, it is acknowledged that registration error has not been avoided entirely. Establishing the correct rotation around the long axis of the LV remains a significant challenge even with the use of coronary ostia and other anatomical landmarks as guidance. Change in the shape of the LV between the time of electrophysiology procedures and imaging are expected due to differences in the loading conditions, which represents an additional challenge, as do absolute limitations in the accuracy of the localization of EGM signals. The degree to which registration error affects the accuracy of results is difficult to quantify and is not accurately described by surface distance between EAMS and imaging data. There is no consensus regarding the optimal threshold to apply in order to accurately identify scar on LGE-CMR (25). The challenge of quantitatively assessing scar and fibrosis are compounded by differences in imaging parameters and contrast administration protocols, which will affect degree of hyperenhancement of tissue. The absence of standard histological data from this study due to the destructive nature of the EACI process is a further limitation of the current study and prohibits formal validation of the imaging data. Common to most large animal pre-clinical studies, this study included a relatively small number of animals, in consideration of minimizing the use of animals in experiments and cost. Despite these limitations, the comparison between LGE-CMR and high density electrophysiological mapping data demonstrate that the imaging acquired and processing strategy reliably identified electrophysiologically relevant tissue in this model.

## Conclusions

The structural characteristics of the tissue demonstrating diastolic activation during reentrant VT-HC, including non-transmural scar, mildly decreased tissue thickness and steep gradients in tissue thickness, are likely to promote functional conduction block which is demonstrated to be an important mechanism underlying the VT-HC observed in this model. The late diastolic segment of activation during VT-HC is closest to steep gradients in tissue thickness which may be a contributory factor to the maximal wavefront slowing seen in this region. The characterization of the myocardial substrate for post-MI scar mediated VT-HC could form the basis for imaging criteria to define ablation targets during future trials.

## Data Availability Statement

The raw data supporting the conclusions of this article will be made available by the authors, without undue reservation.

## Ethics Statement

The animal study was reviewed and approved by Animal studies complied with French law and were performed at the Institut de Chirurgie Guidée par l'image (IHU), Strasbourg, France. The experimental protocol was approved by the Local and National Institutional Animal Care and Ethics Committee.

## Author Contributions

JW: conceived and designed study, analyzed data and prepared first draft and subsequent modifications of the manuscript. RN and SR: designed and implemented CMR protocols, assisted in acquisition of imaging and contributed to analysis of data. SK: contributed to experimental design, assisted with acquisition of electroanatomic mapping data, and contributed to analysis of data. AC: key contribution to analysis of electrophysiologic data. TA: planning and acquisition of electrophysiological data. JC, MW, and JS: electrophysiologist who acquired electro-anatomic mapping data. RK: developed computational tools for registration of imaging and electrophysiologic data. BM, CRi, SM, and LC: development of VA-ECMO protocol for hemodynamic support, delivery of hemodynamic support during electrophysiology studies. TI and JH: CMR reader. JV and MA: development of cryomicrotome imaging protocol and processing of tissue to acquire cryomicrotome imaging and approved submitted version of manuscript. SW, LO'N, RM, HC, RR, and MO'N: involved in acquisition of CMR and electrophysiological data. RM: repeat segmentation of 3d LGE CMR imaging for inter-observer reproducibility. HC, RR, and MO'N: funding holder for study. SW, LO'N, RM, HC, RR, MO'N, CT, EA, SN, and MB: planning and study design. RR and MO'N: supervisory oversight of project. CT, EA, SN, MB, HC, RR, MO'N, RM, SW, LO'N, TI, JH, BM, CRi, SM, LC, CRo, RK, JC, MW, JS, TA, AC, SK, RN and SR: substantial modifications to previous drafts of manuscript and approved submitted version of manuscript. TA, JC, MW, JS, BM, CRi, SM, LC, CRo, TI, JH, RK, SW, LO'N, RM, CT, EA, SN, MB, HC, RR, and MO'N: analysis and interpretation of data. All authors contributed to the article and approved the submitted version.

## Funding

This work was supported by a Medical Research Council UK Clinical Research Training Fellowship (grant code MR/N001877/1) which funded JW; the Health Innovation Challenge Fund (HICF-R10-698), a parallel funding partnership between the Department of Health and the Wellcome Trust, the Wellcome Engineering and Physical Sciences Research Council (EPSRC) Centre for Medical Engineering at King's College London (WT203148/Z/16/Z); EPSRC grant (EP/R010935/1); National Institute for Health Research (NIHR) Biomedical Research Centre award to Guy's and St Thomas' National Health Service (NHS) Foundation Trust in partnership with King's College London, and by the NIHR Healthcare Technology Co-operative for Cardiovascular Disease at Guy's and St Thomas' NHS Foundation Trust. The views expressed are those of the author(s) and not necessarily those of the NHS, the NIHR or the Department of Health. The CardioHelp machine and all ECMO consumables were donated by Maquet, Getinge Group without conditions on their use. The Precision and Claris system and all EP mapping consumables were donated by Abbott without conditions on their use. This research was funded in whole, or in part, by the Wellcome Trust [Grant Number WT203148/Z/16/Z]. For the purpose of open access, the author has applied a CC BY public copyright licence to any Author Accepted manuscript version arising from this submission. This project was supported by the National Institute of General Medical Sciences of the National Institutes of Health [Grant Numbers P41 GM103545 and R24 GM136986]. SW was supported by British Heart Foundation [Grant Number FS/20/26/34952].

## Conflict of Interest

The CardioHelp machine and all ECMO consumables were donated by Maquet, Getinge Group without conditions on their use. The Precision and Claris system and all EP mapping consumables were donated by Abbott without conditions on their use. The authors declare a potential conflict of interest and state it below. RN was employed by the company Siemens Healthcare. SK and TA were employed by the company Abbott Medical. The remaining authors declare that the research was conducted in the absence of any commercial or financial relationships that could be construed as a potential conflict of interest.

## Publisher's Note

All claims expressed in this article are solely those of the authors and do not necessarily represent those of their affiliated organizations, or those of the publisher, the editors and the reviewers. Any product that may be evaluated in this article, or claim that may be made by its manufacturer, is not guaranteed or endorsed by the publisher.
