# Supplementary material for: Late Gadolinium Enhancement Cardiovascular Magnetic Resonance Assessment of Substrate for Ventricular Tachycardia With Hemodynamic Compromise
Source: Front Cardiovasc Med. 2021 Oct 26;8:744779. doi: 10.3389/fcvm.2021.744779 (PMC8576410; doi:10.3389/fcvm.2021.744779)
Supplement: Supplementary file 2 [file Data_Sheet_2.pdf]

Supplementary Material

1 Supplementary Data

METHODS

General anaesthesia

Pigs were fasted for 24 h before intervention with free access to water. Premedication was by intramuscular injection of ketamine (20 mg/kg) and azaperone (2 mg/kg) (Stressnil; Janssen-Cilag, Belgium) administered 10 min before induction of anaesthesia with intravenous Propofol (3 mg/kg) and tracheal intubation facilitated with rocuronium (0.8 mg/kg). Pigs were ventilated at a rate of 20 – 26 breaths per minute. Anaesthesia was maintained with inhaled isoflurane at a concentration of 1 – 3%, mixed with 21% Oxygen and air. Intravenous fentanyl was administered for analgesia.

Venous-arterial extra corporeal membrane oxygenation (VA-ECMO)

Hemodynamic support was instituted as follows: Percutaneous femoral access was established using 2-dimensional ultrasound to guide the percutaneous insertion of a 5Fr hydrophilic Micropuncture radial access kits using a Seldinger technique (CheckFlo Performer Introducer set, Cook Medical Inc, Bloomington, IN, USA): two in the right femoral artery, two in the right femoral vein; one in the left femoral artery and one left femoral vein. On the right, one arterial sheath was upgraded to a 9Fr haemostatic sheath and one arterial sheath was upgraded to a 9.5Fr Agilis NxT steerable introducer (Abbott, St Paul, MN). The right sided femoral venous sheaths were upgraded to 7Fr and 8Fr haemostatic sheaths respectively. The left sided femoral arterial puncture site was serially dilated using an 8Fr followed by 12Fr dilator prior to inserting a 15Fr steel reinforced extra-corporeal membrane oxygenation (ECMO) return cannula (Maquet, Rastatt, Germany) which was flushed using 100ml of heparinised saline and then clamped. The left sided femoral venous puncture site was

serially dilated using an 8Fr followed by 12Fr followed by 14Fr dilator prior to inserting a 21Fr steel reinforced ECMO venous access cannula (Maquet) which was flushed using 100ml of heparinised saline and then clamped. ECMO cannulae were sutured in place. A quad lumen central venous catheter (Multicath Expert, Vygon, Swindon, UK) was inserted into the right or left internal jugular vein. An arterial cannula was placed in the left or right carotid artery to monitor systemic arterial blood pressure via the Claris WorkMate electrophysiology recording system (Abbott, St Paul, MN, USA).

Haemodynamic support was prophylactically instituted at the start of the procedure in the form of VA-ECMO delivered using a Maquet Cardiohelp machine. The Cardiohelp circuit was primed with 2L of warmed and heparinised normal saline. A systematic procedure was followed to remove air bubbles from the circuit[1] which included a flow sensor and bubble detector on the venous access line. On completion of the bubble removing procedure the venous and arterial lines were clamped and the tubing connecting the arterial and venous limbs of the circuit was cut. The return circuit was then connected to the arterial ECMO cannula and the venous limb of the ECMO circuit was connected to femoral venous ECMO cannula using an underwater seal. Four clamps on both sides of the tubing connection in each limb of the circuit were removed and flow in the circuit commenced immediately at the minimum flow rate of 0.5l/min. The circuit tubing was secured to the table using waterproof duct tape. Unfractionated heparin (100 U/kg IV) was given as a bolus after sheaths placement followed by 40 to 50 U/kg/h to maintain activated clotting time of 180 to 250s. Regular venous and arterial samples were collected and analysed using an i-STAT® (Abbott, Princeton, NJ) point of care blood sample analysis machine, which included blood gas analysis, activated clotting time, chemistry and haemoglobin. VA-ECMO flow was titrated to the minimum flow rate required to maintain a mean arterial pressure (MAP) of 70mmHg throughout the procedure. The selective α_1-adrenergic

receptor agonist phenylephrine was administered intravenously to provide additional vasopressor support as required. The vascular access configuration is shown in supplementary figure 1.

Electrophysiology study additional details

12 lead surface ECG was recorded throughout all cases with standard filter settings

A sensor-enabled Abbott FlexAbility™ ablation catheter was advanced to the aorta via the right femoral artery and used to acquire aortic root and coronary ostia geometry prior to gaining retrograde left ventricular (LV) access across the aortic valve. A multipolar mapping catheter (HD Grid™ (1mm electrodes or LiveWire™ duo-deca (Abbott, Chicago, IL)) was advanced through an Agilis sheath via the aorta to acquire LV endocardial geometry.

All electrophysiology studies were conducted using a Precision™ EAMS (Abbott, Chiocago, IL) and a WorkMate Claris™ recording system with pacing stimulator. The right femoral venous access was used to place a decapolar catheter (Livewire, Abbott) in the coronary sinus and a 6Fr pentapolar catheter (5-5-5-152mm spacing) in the right ventricular apex (Boston Scientific, Marlborough, MA, USA) such that the proximal electrode was free in the inferior vena cava (IVC).

During activation mapping strict settings were applied to the Automap function within the Precision™ electro-anatomic mapping system (EAMS, Abbott, Chicvago, IL) (Score = 85; speed limit = 10mm/s; distance 1mm; enhanced noise rejection: off).

When VT was induced, activation maps were acquired using the initial deflection seen on any lead of the surface ECG leads as the timing reference and the same Automap settings as above. Each individual bipolar and unipolar EGM from the mapped VTs and maps during pacing were subsequently reviewed offline and the activation time reassigned when necessary. Once a sufficiently high-density activation map had been collected and if the VT was sustained, an attempt was made to reset the tachycardia with single, synchronised extra-stimuli and then to entrain the tachycardia from

a site demonstrating diastolic activation. Following spontaneous or pace-termination, VT induction and mapping was subsequently repeated. In the event of VF, a 200J biphasic shock was administered. Geometry was adjusted and/or reacquired following defibrillation or shift due to subject movement.

EGM data and model point cloud from each paced map and each VT was exported from the EAMS and then imported into Matlab using a custom written, publicly available EAMS data reader (https://openep.io/).

Cardiovascular magnetic resonance imaging data analysis

Each 3D LGE-CMR scan was manually segmented by a single observer within Seg3D2 (University of Utah, Utah, USA[17]). The segmented myocardium was thresholded for scar signal intensity (SI_{scar}) at 60% and intermediate SI ($SI_{intermediate}$) at 40% of the highest SI within the segmentation.

Image quality

Objective parameters of image quality were assessed as follows: Signal-to-noise ratio (SNR) and contrast-to-noise ratio (CNR) were estimated using mean signal within blood, remote (healthy) myocardium and scar and standard deviation of signal within lung. A subjective assessment of image quality and comparison with 2D clinical standard imaging according to pre-defined parameters was undertaken by two experienced, SCMR level 3 accredited readers (JH/TI). The 3D bSSFP LGE and 2D LGE magnitude images were scored on a Lickert scale of 1 – 4 according to image sharpness, scar contrast, quality of nulling of the healthy ventricular myocardium, the complexity evident in the scar pattern and the overall scan quality. The readers assigned a score of 1 for non-diagnostic imaging, 2 for major artefact but diagnostic imaging, 3 for minor artefact but diagnostic imaging and 4 for no significant artefact. Example imaging that was assigned scores 2 – 4 are shown in the supplementary figure 2).

Tissue thickness gradient assessment

Gradient of tissue thickness was calculated by decimating the *in-vivo* derived mesh to 50% of its original size within Paraview. Data was imported into Matlab, and a radial basis function was applied to interpolate the decimated tissue thickness from measured points to each node of the triangulated LV endocardial shell. The gradient of the interpolated thickness was calculated to estimate local tissue thickness gradient.[2]

Regions of steep transition in tissue thickness were considered to be those with a gradient in tissue thickness of above 1 (spatial rate of change in tissue thickness, therefore no units) and tissue thickness between the median thickness of isthmus locations and healthy tissue (5.5 -8.2mm). The geodesic distance between locations on the endocardial shell was calculated using a Matlab based implementation of the Dijkstra algorithm.[3]

Segmentation approach

Papillary muscles were included in the LV blood pool segmentation if there was contrast between the papillary muscle body and the endocardial surface, and they were included in the myocardial segmentation if there was no visible blood pool between the muscle body and the endocardial surface. This strategy was selected to reflect as closely as possible the endocardial surface that would have been in contact with the electrophysiology catheters within the left ventricle.

Generation of surface meshes from in-vivo CMR imaging

Volumetric meshes were generated from the myocardium, blood pool, scar, HT and aorta and coronaries segmentations within Tarantula (www.meshing.org). Surface mesh files (vtk format) were generated from exported volumetric meshes within the open source Meshalyzer package (https://github.com/cardiosolv/meshalyzer) which were subsequently imported into Matlab and processed to generate a surface mesh containing endocardial and epicardial surfaces, scar and aorta in

the Digital Image Fusion (DIF) format for import into the Precision electroanatomic mapping system (EAMS).

Episcopic auto-fluorescence cryomicrotome imaging

Following euthanasia each heart was embedded in 5% carboxymethylcellulose sodium solvent (Brunschwig Chemie, The Netherlands) and Indian ink 5% (Royal Talens, The Netherlands), then frozen at -20°C. The frozen, embedded hearts were placed in an Imaging Cryomicrotome (IC). Within the IC, hearts were serially sectioned in an approximately short axis orientation at a slice thickness of 60µm. The sample was illuminated with a cluster of power light emitting diodes (Luxeon V, Star, Royal Blue, Philips Lumileds Lighting, USA). Specific excitation (480nm) and emission (577nm) light bandwidths were selected through the use of optical filters (Chroma Technology Corp., Bellows Falls, VT, USA). After each slice, the remaining bulk surface was imaged with fluorescent optical surface imaging using a 4096 x 4096 pixel digital camera (Apogee Alta U-16, USA) equipped with a variable focus lens (Nikon, 70 – 180mm, The Netherlands), providing an in-plane resolution of 30µm, as previously described. [4] Images were processed and segmented, then thresholded at an automatically identified SI threshold to identify enhanced regions corresponding to scar.

Raw EACI data (a stack of z co-registered images with m rows and n columns) was processed to correct for a visually apparent bias in the signal intensity across the image due to angle of incidence of the illuminating light when the tissue was imaged and to remove a series of vertical striations representing artefact from the tissue cutting process.

Bias field removal was performed independently for each image stack, assuming a constant bias field for each image slice in the stack. All images were assumed to have the same bias field. The bias field appeared to show a gradient only in the vertical direction in the images. A single image, I, from the

top or bottom of the stack that contained no tissue (and therefore no autofluorescence) was manually selected. From image *I*, a vector, $\boldsymbol{v}$, of length *m,* containing the mean row-wise SI was calculated:

$$v_i = \frac{1}{n}\sum_{j=1}^{n} I_{ij} \quad (1)$$

Where v_i is a vector of length m and I is the matrix of signal intensity values within an image without any autofluorescence of dimensions m x n.

Linear regression was performed to establish the line of best fit:

$$y_i = \beta \cdot i + \alpha \quad (2)$$

Where β is the gradient of the line of best fit, α is the intercept and i is the row index

Each row of pixels in each image in the image stack was then normalized via the corresponding value of the line y_i at the same row index, i:

$$I_{ij}\ assigned\ SI = \frac{I^{\wedge}{}_{ij}}{y_i} \quad (3)$$

A spectral masking process was used to remove the vertical striations. Each image in the stack was fast-Fourier transformed (FFT) in two dimensions in Matlab to give the Fourier-transformed frequency spectrum, $\hat{I}$. The zero-frequency component of $\hat{I}$ was then shifted to the centre of the matrix. High frequency components corresponding to the vertical striations in the original image were set to zero by multiplying thee shifted Fourier transformed image, $\hat{I}$, by a spectral mask, *Q.* Multiplication by the spectral mask removed a central band in the frequency domain, the width of which was adjusted manually achieve adequate striation removal performance without introducing aliasing in the inverse-transformed image. The centrally shifted, spectrally masked, fast-Fourier

transformed image was then shifted back to the image origin and inverse Fourier transformed, following which the real components of this result gave the de-lined image.

Each series of EACI images were imported into Seg3D2 (University of Utah, Utah, USA (seg3D reference) and manually segmented by a single observer within Seg3D2. Papillary muscles were included in the LV blood pool segmentation if there was contrast between the papillary muscle body and the endocardial surface, and they were included in the LV myocardial segmentation if there was no visible blood pool between the muscle body and the endocardial surface. This strategy was selected to reflect the strategy used to segment the *in-vivo* CMR scans.

Binary segmentations of the LV blood pool, myocardium and aorta/coronary vessels were saved. A ROI within the basal myocardial segmentation, remote from the vascular territory of the infarct and the region visually identified as remote from the infarction, and a ROI in a region of increased SI within the vascular territory of the infarct were manually segmented. The threshold for scar was automatically calculated within Matlab and selected per dataset in order to identify the SI threshold on histology which gave a volume with closest correspondence to the *in-vivo* scar volume.

Within Seg3D2, the threshold filter was used to select voxels with a SI above the lower boundary threshold for scar. A connected component filter with seeds placed in clear regions of scar was applied. Additional manual deletion of voxels that were deemed to be enhanced but remote from scar was performed. This was typically restricted to the edges of tissue which frequently demonstrated enhancement, as shown in supplementary figure 3. No voxels were added to the scar segmentation.

RESULTS

2D versus 3D LGE CMR imaging

3D LGE-CMR was acquired in mid-diastole with a total imaging time of 50+/-12 minutes compared to 2D LGE-CMR which was acquired in mid-diastole with a total imaging time of 7.0+/-2.1 minutes ($p<0.001$). Objective and subjective measures of image quality were higher in the 3D compared to the 2D LGE CMR imaging (figure 3SM). SNR_{scar}, SNR_{blood} and $CNR_{scar-healthy}$ was higher in 3D LGE-CMR imaging than the 2D LGE imaging ($p<0.001$), while $CNR_{scar-blood\ pool}$ was higher in the 2D imaging ($p=0.039$), possibly reflecting the higher FA chosen for the 3D imaging. $SNR_{healthy}$ was lower in the 3D LGE-CMR than the 2D LGE imaging ($p<0.001$), indicating more effective nulling of the myocardium. Subjectively, the 3D LGE-CMR images were sharper (4.0 vs 2.5, $p=0.026$), had better contrast (4.0 vs 3.0, $p=0.024$), clearer demonstration of scar complexity (4.0 vs 2.5, $p=0.038$) and greater overall scan quality (4.0 vs 2.5, $p=0.041$) compared to the 2D LGE imaging. These results are shown in supplementary figure 4.

Inter- and intra-observer reproducibility of segmentation of 3D LGE CMR imaging

Intra-observer and inter-observer reliability of the manual segmentation was assessed for all the 3D LGE CMR imaging. One observer (JW) segmented myocardium and blood pool at two time points, separated by at least three months. A second observer (RM) segmented the same imaging. The intra-class correlation coefficient (ICC) was calculated between observations from the same observer and between measurements from different observers using a two-way mixed model assessing for absolute agreement between observations. In addition, Dice-Similarity Coefficient (DSC) was calculated to assess for agreement between volumes segmented between repeat segmentations from the same observer and between observers.

For average measures, the ICC between observations of myocardial volume from the same observer and between observers demonstrated excellent agreement in each case (intra observer ICC = 0.91, p = 0.003; inter observer ICC = 0.96, p<0.0001) and the ICC between observations of blood pool volume from the same observer and between observers demonstrated excellent agreement in each case (intra observer ICC = 0.97, p<0.0001; inter observer ICC = 0.95, p<0.0001). These results indicate that the manual segmentation method used to analyze the 3D-LGE imaging produces reproducible estimates of myocardial and blood pool volumes.

Mean DSC was within the range of good agreement for all measures (intra-observer myocardium: 0.92(+/-0.02); intra-observer blood pool: 0.95(+/-0.02); inter-observer myocardium: 0.88(+/-0.04); inter-observer blood pool 0.95(+/-0.01)) demonstrating consistent assignment of tissue as myocardium and blood pool within the same imaging.

2 Supplementary Figures and Tables

2.1 Supplementary Figures

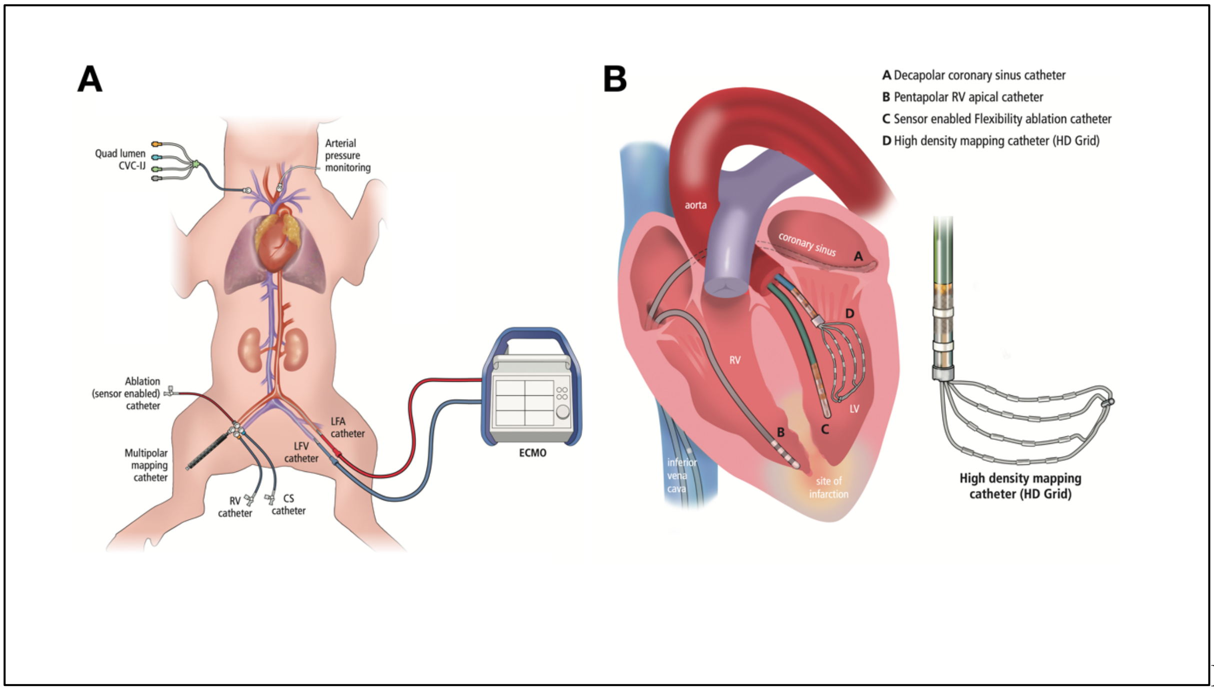

Figure

Supplementary figure 1: Vascular access and catheter placement during electrophysiology study.

Panel A: Vascular access established prior to EP study showing venous-arterial extra-corporeal membrane oxygenation (VA-ECMO) catheters in the left femoral vessels and EP catheters in the right femoral vessels, central venous access in the right internal jugular and arterial access via the left carotid artery. Panel B: Diagnostic EP catheter placement in the heart including decapolar catheter in the coronary sinus (A) and pentapolar catheter in the RV apex (B) via the inferior vena cava and sensor enabled Flexability ablation catheter (C) and high density multipolar mapping catheter (D) advanced into the left ventricle via a retrograde aortic approach.

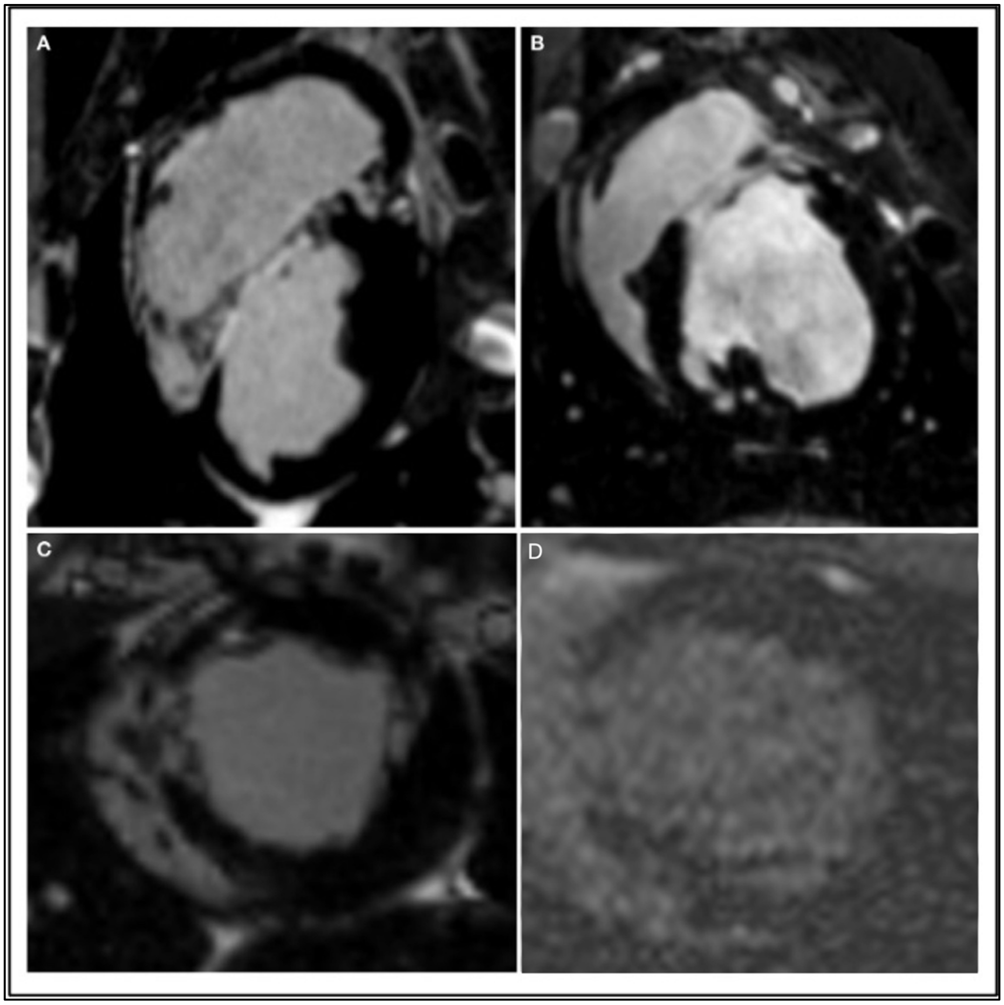

Supplementary figure 2: Examples of imaging assigned a range of scores for overall image quality on the Lickert scale. Panel A: Acquisition orientation 3D bSSFP imaging from animal 1 in an example of imaging assigned 4 for overall image quality. Panel B: Short axis multi planar reconstruction (MPR) of 3D bSSFP LGE imaging from animal 3, assigned 4 for overall image quality. Panel C: Short axis MPR of 3D bSSFP LGE imaging from animal 2, assigned 3 for overall image quality. Panel D: 2D LGE imaging from animal 7, assigned 2 for overall image quality.

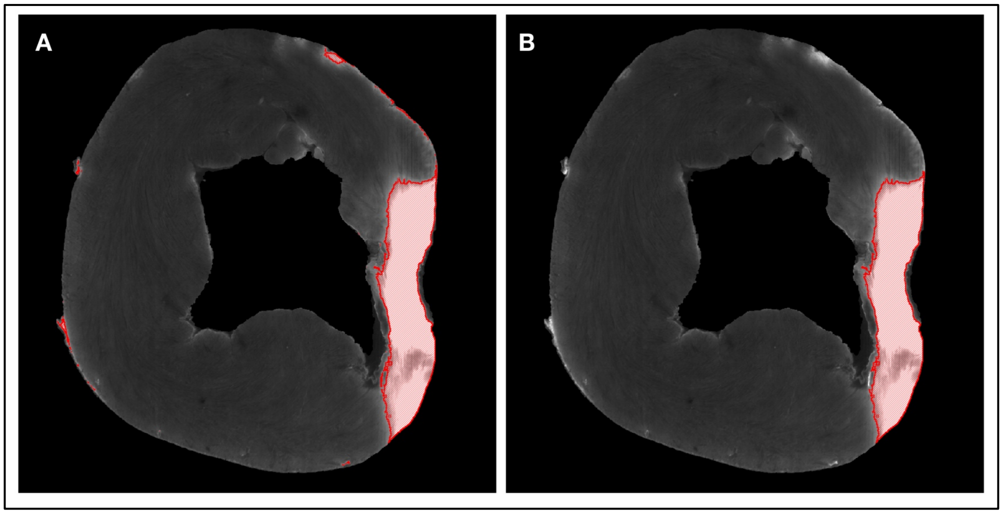

Supplementary figure 3: Example of manual deletion of enhanced edges in histology tissue. *Example from pig 1* Panel A: Short axis (SAX) slice of episcopic autofluorescent cryomicrotome imaging (EACI) of the left ventricle (LV). There is an enhanced and thinned region in the septum. Unadjusted intensity based threshold applied to EACI demonstrates inclusion of small portions of the tissue edge as well as epicardial blood vessels in the scar mask shown in red. Panel B: Same SAX slice as shown in panel A with scar mask superimposed, which is the same scar mask shown in panel A following application of a connected component filter with seeds placed in the scar region which was visually identified in the vascular territory of the occluded coronary vessel.

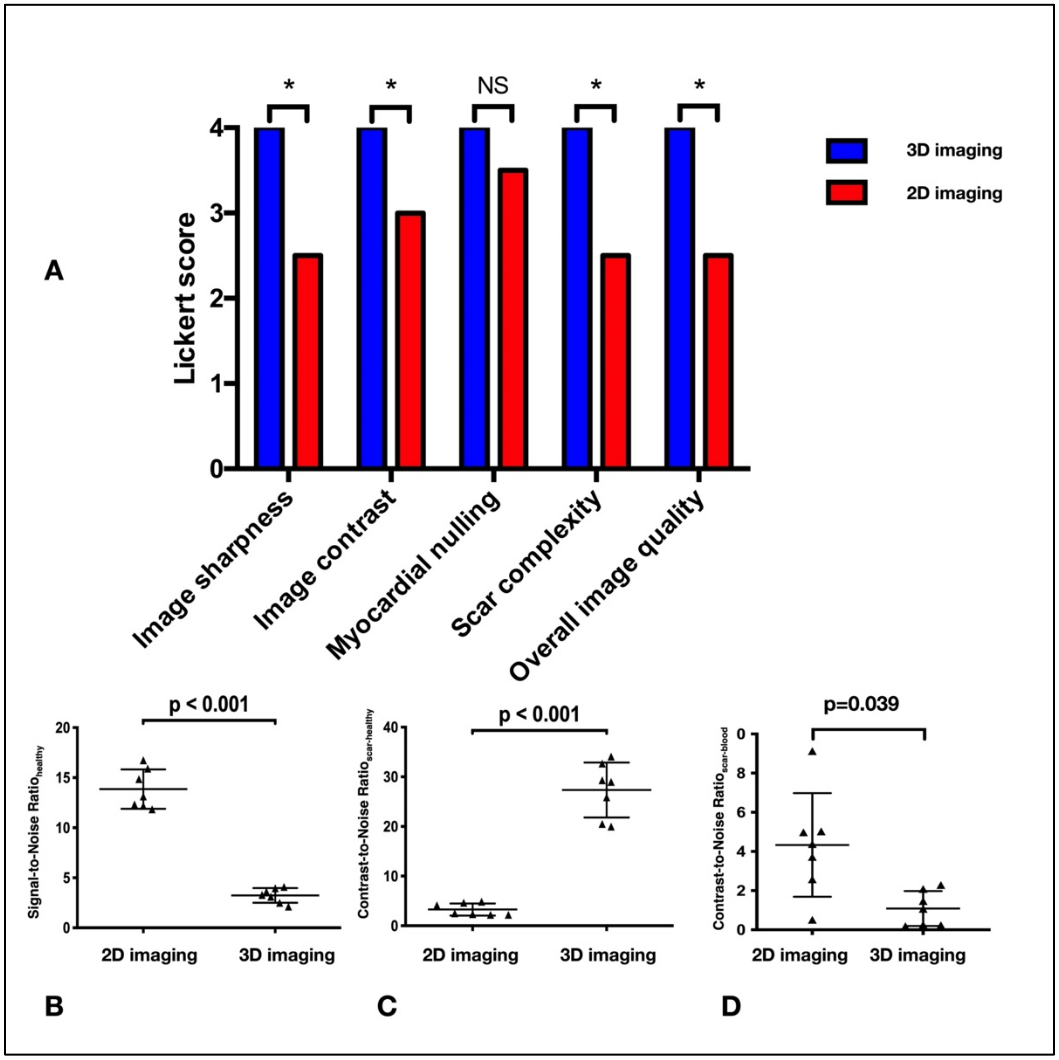

Supplementary figure 4: Subjective and objective assessment of image quality compared between 2D and 3D LGE-CMR imaging. Panel A: Qualitative comparison in image quality between 2D LGE-CMR following contrast bolus and 3D LGE-CMR acquired under conditions of contrast steady state (CSS). **Panel B:** Objective comparison between signal to noise (SNR) in healthy tissue between 2D LGE-CMR and 3D LGE CMR. **Panel C:** Comparison between contrast to noise ratio (CNR) between scar and healthy tissue in 2D LGE-CMR and 3D LGE-CMR. **Panel D:** Comparison of CNR between scar and blood pool in 2D LGE-CMR and 3D LGE-CMR.

3 References

1. Bishop M, Rajani R, Plank G, Gaddum N, Carr-White G, Wright M, O'Neill M, Niederer S. Three-dimensional atrial wall thickness maps to inform catheter ablation procedures for atrial fibrillation. *Europace*. 2016;18:376–383.

2. Masè M, Ravelli F. Automatic reconstruction of activation and velocity maps from electro-anatomic data by radial basis functions [Internet]. In: 2010 Annual International Conference of the IEEE Engineering in Medicine and Biology. IEEE; 2010. p. 2608–2611.

3. Dijkstra EW. A Note on Two Probles in Connexion with Graphs. *Numer Math*. 1959;1:269–271.

4. van Horssen P, van den Wijngaard JPHM, Brandt MJ, Hoefer IE, Spaan JAE, Siebes M. Perfusion territories subtended by penetrating coronary arteries increase in size and decrease in number toward the subendocardium. *Am J Physiol Circ Physiol*. 2013;306:H496–H504.
